# Studying Hospitalizations and Mortality in the Netherlands: Feasible and Valid Using Two-Step Medical Record Linkage with Nationwide Registers

**DOI:** 10.1371/journal.pone.0132444

**Published:** 2015-07-06

**Authors:** Elske Sieswerda, Anna Font-Gonzalez, Marcel G. W. Dijkgraaf, Ronald B. Geskus, Richard C. Heinen, Helena J. van der Pal, Flora E. van Leeuwen, Huib N. Caron, Leontien C. Kremer, Johannes B. Reitsma

**Affiliations:** 1 Department of Pediatric Oncology, Emma Children’s Hospital / Academic Medical Center, Amsterdam, the Netherlands; 2 Clinical Research Unit, Academic Medical Center, Amsterdam, the Netherlands; 3 Department of Clinical Epidemiology, Biostatistics and Bioinformatics, Academic Medical Center, Amsterdam, the Netherlands; 4 Department of Medical Oncology, Academic Medical Center, Amsterdam, the Netherlands; 5 Department of Epidemiology, Netherlands Cancer Institute, Amsterdam, the Netherlands; 6 Julius Center for Health Sciences and Primary Care, University Medical Center Utrecht, Utrecht, the Netherlands; INRCA, ITALY

## Abstract

In the Netherlands, the postal code is needed to study hospitalizations of individuals in the nationwide hospitalization register. Studying hospitalizations longitudinally becomes troublesome if individuals change address. We aimed to report on the feasibility and validity of a two-step medical record linkage approach to examine longitudinal trends in hospitalizations and mortality in a study cohort. First, we linked a study cohort of 1564 survivors of childhood cancer with the Municipal Personal Records Database (GBA) which has postal code history and mortality data available. Within GBA, we sampled a reference population matched on year of birth, gender and calendar year. Second, we extracted hospitalizations from the Hospital Discharge Register (LMR) with a date of discharge during unique follow-up (based on date of birth, gender and postal code in GBA). We calculated the agreement of death and being hospitalized in survivors according to the registers and to available cohort data. We retrieved 1477 (94%) survivors from GBA. Median percentages of unique/potential follow-up were 87% (survivors) and 83% (reference persons). Characteristics of survivors and reference persons contributing to unique follow-up were comparable. Agreement of hospitalization during unique follow-up was 94% and agreement of death was 98%. In absence of unique identifiers in the Dutch hospitalization register, it is feasible and valid to study hospitalizations and mortality of individuals longitudinally using a two-step medical record linkage approach. Cohort studies in the Netherlands have the opportunity to study mortality and hospitalization rates over time. These outcomes provide insight into the burden of clinical events and healthcare use in studies on patients at risk of long-term morbidities.

## Introduction

Survivors of childhood cancer are an example of a patient group that has an increased risk of long-term morbidity and mortality [1–5]. To study clinical events in these kinds of patient groups, it is possible to assess outcomes of interest in a study cohort periodically through clinical assessments. This approach is very time-consuming and costly, due to the relatively low absolute frequency of unfavorable health conditions and the potentially long duration between risk factor (for example: cancer treatment) and clinical event (such as treatment-induced health problems). To determine whether certain health problems occur more frequently than expected, an appropriate (unexposed) reference population is necessary. Clinical follow-up of such a reference population will generate additional costs.

For these reasons, it is appealing to use readily available data such as data from national administrative registers and to link a study cohort to these registers. Such medical record linkage studies allow examination of the relation between detailed information on risk factors of the cohort and the clinical events that are routinely registered in administrative registers. The registration of data from a complete population makes it additionally possible to compare outcomes to an appropriate reference population. In the Netherlands, there is potential electronic access to an administrative register containing near-complete and high-quality national hospitalization data from 1995 onwards. As in other countries, the health care system in the Netherlands in general does not use a unique person identification number. Registration of hospitalizations of individuals is based on gender, date of birth and postal code at the date of discharge. This level of anonymity previously limited longitudinal identification of hospitalizations in medical record linkage studies due to moving (change in registered postal code).

In 2003 it became possible to link information from the Municipal Personal Records Database (Gemeentelijke Basisadministratie; Dutch acronym: GBA) to the Hospital Discharge Register (Landelijke Medische registratie; Dutch acronym: LMR) [6]. GBA is a nationwide register in which current and previous addresses of all Dutch citizens are recorded. GBA can now be used to link multiple hospitalizations from LMR over time to one individual. This opens the door for cohort studies to assess long-term hospitalizations of their patients. Hospitalization rates over time provide a measure of burden of disease on individuals and on healthcare resources [7, 8]. However, it is essential to be aware of the feasibility and potential biases of such studies.

The objective of this study was therefore to determine the feasibility and validity of studying mortality and hospitalizations over time in a clinical cohort using a two-step medical record linkage approach. To study this, we linked a cohort of survivors of childhood cancer from the Emma Children’s Hospital/Academic Medical Center (EKZ/AMC) with (1) GBA and (2) LMR. We will describe the key technical steps in this linkage project, the quality of record linkage, and discuss potential strengths and limitations.

## Methods

### Registers

The EKZ/AMC Childhood Cancer Survivor cohort is an on-going, single-center, cohort study of survivors of childhood cancer with the goal to study the risk of unfavorable health conditions associated with their previous treatment [9]. Experienced data managers, supervised by a pediatric oncologist, are responsible for enrolment of eligible patients, data collection and updates, using structured protocols and an extensive data dictionary. Baseline primary cancer treatment characteristics (i.e. start and end date of treatment and whether or not treatment included any surgery, radiotherapy, chemotherapy and/or other therapy) are complete for 97.7% of survivors.

GBA is an administrative database in which municipalities register demographic information, such as the Dutch citizen service number (Dutch acronym: BSN), gender, birth, address and postal code, country of birth, marital status and death of their residents. One of its goals is to provide data for population statistics. It contains electronic information of all permanent residents in the Netherlands since October 1, 1994. When an individual changes address, this is registered in GBA. In this way it is possible to get signals from GBA about the (change in) address once a person has been identified within GBA [10]. A study about the quality of recording postal code in GBA showed that for 98 to 99% of the Dutch population the right address is present [11]. Linkage of individuals with GBA can be based on the unique BSN or on the combination of date of birth, gender and postal code at a certain reference date [10].

LMR is an administrative register that contains electronic information on hospital admissions of almost all hospitals in the Netherlands from 1995 onwards, with a coverage of >98.9% until 2004 and 96.7% in 2005 [12]. After 2005 the coverage has decreased due to administrative changes in the Dutch health care system. One goal of LMR is to provide data for population statistics. Coding of discharge diagnoses is performed by hospitals according to a uniform coding handbook [11]. Regular validity checks are done to ensure the quality of the data and a study about the quality of LMR data showed high quality data. Personal information, dates of hospital admission and discharge were correct in 99% of hospitalizations and principal diagnoses (as compared with medical record review by medical specialists) were correct in 84% of hospitalizations [13].

Hospitalization registration includes date of birth, gender and postal code of the hospitalized person at the date of discharge, but it does not contain a unique person identifier. Therefore, linkage of hospitalizations to individuals is based on the combination of gender, date of birth and postal code. This creates two potential problems: (1) the combination of these three variables does not always belong to one unique person and (2) admissions after a person moves cannot be identified, as the new address is needed for linkage.

The first problem of non-unique hospitalized persons cannot be solved as long as Dutch policy makers do not allow for a unique identifier in LMR. On average, 84% of hospitalizations in the Netherlands are attributable to one unique person based on date of birth, gender and postal code [11]. The second problem now can be solved using information from GBA. Using GBA it is possible to define history of postal codes of individuals and to link hospitalizations at a certain date to the person with a postal code at that specific date. Thus, by using GBA it is now possible to link (multiple) hospitalizations to persons who moved in the period after or between hospitalizations. In a report about linkage between GBA and LMR, it was found that more than 97% of the uniquely linked hospitalizations were linked correctly [14].

For the current study we used GBA files for the years 1995 to 2008 and LMR files for the years 1995 to 2005, provided by Statistics Netherlands (Centraal Bureau voor de Statistiek; Dutch acronym: CBS).

### Study population

Inclusion criteria for the study cohort were as follows: diagnosed with a primary cancer diagnosis below age 18; primarily treated in the EKZ/AMC; diagnosed between January 1st 1966 and January 1st 1999; survival at least 5 years since primary cancer diagnosis. Because we could not electronically link individuals who died before 1995 to GBA, we excluded these patients from the current study. We made special attempts to extract identification data from our cohort to enable the record linkage to GBA: the unique BSN and the combination of gender, date of birth and postal code (with a reference date of the postal code). If a postal code at an additional reference date was available for a survivor within the cohort, this was also extracted.

### Ethics statement

Written informed consent was obtained from all childhood cancer patients treated in the EKZ/AMC. The Institutional Review Board of the EKZ/AMC in Amsterdam reviewed and approved the data collection for our cohort register and the study was deemed as evaluation of patient care and was therefore exempt from the need for ethical approval.

We performed this study according to the Federa (Council of the Federation of Medical Scientific Societies) Code of Conduct for the Use of Data in Health Research [15]. This Code was developed in 1995 and revised in 2002–2003 on the basis of the European Data Protection Directive and its implementation in the Dutch Act on the Protection of Personal Data. After medical record linkage of the cohort to national registers, the data did not include directly identifiable variables anymore. We analysed data anonymously and made sure that results would not disclose individual data. For more information on legal matters and logistics, see S1 Methods.

### Dutch sample of the general population as reference population

For computer efficiency reasons, we randomly selected 20 reference persons at maximum per survivor with the same year of birth and gender as all individual survivors that could be linked to GBA. Selecting even more reference persons would considerably increase computer time, but would hardly lead to any gain in the precision of the effect measures of interest. The starting date of follow-up in reference persons was set to the same starting date of follow-up in the corresponding cancer survivor (i.e. five year after the date of primary cancer diagnosis of the corresponding survivor). Persons in the reference population could only be sampled once and had to be alive, living and registered in the Netherlands after the corresponding date of five-year survival and between January 1, 1995 and January 1, 2006.

### Linkage of a clinical cohort to the GBA

In the first linkage step Statistics Netherlands linked a data file of our study cohort with a data file from GBA, based on a specifically developed and validated record linkage protocol involving three potential linkage options (See S1 Fig for the key steps in the record linkage process).

Linkage to GBA was based on (a) the unique Dutch citizen service number when available in a 1–1 deterministic linkage procedure.

The remaining survivors were linked based on deterministic linkage with the combination of date of birth, gender and (b) a postal code at a first reference date or (c) a postal code at a second reference date (date when the postal code was verified to be correct). The postal code included the four numbers and two digits used in the Netherlands. Survivors who were not identified within GBA were excluded from further analyses. Information of death during the study was retrieved from GBA and thus available once as a person was identified in GBA.

### Extraction of hospitalization data based on link between GBA and LMR

The second linkage step was the extraction of hospitalizations from a LMR data file. This was done for all survivors identified in GBA and the matched reference persons. We retrieved all hospitalizations over time with a date of discharge during a period in which a person was unique based on the combination of date of birth, gender and four number postal code in GBA. Around 16% of the full combination of these first three variables is not unique within GBA due to another person with the same combination of variables (administrative twin). There could thus be missing periods in individuals due to (temporary) non-uniqueness. Accrual of unique follow-up time of the individual linkage period began at the first date a person was unique in GBA since the (corresponding) date of five-year survival or January 1st, 1995, whichever came latest. Accrual of unique follow-up time of the individual linkage period ended at the date of death, date of emigration, date a person was not unique anymore or January 1st, 2006, whichever came first. When multiple unique periods were present within a person (due to a period of non-uniqueness), we summed up the follow-up time of the unique periods to define the total unique follow-up time for this individual. We excluded the days spent in hospital from the time at risk of such an individual.

### Quality of linkage process

We explored the quality of the linkage process by assessing the potential threats to validity of the two steps in the linkage process. First, we examined whether the loss of survivors during linkage with GBA and the loss of hospitalization information due to individuals having a non-unique combination of gender, date of birth and postal code at the date of discharge could be considered a random process. This was done by comparing distributions of important clinical and (for survivors) treatment characteristics (1) between survivors linked and survivors not linked to GBA, (2) between the survivors or reference persons contributing to unique follow-up time in the study and those who did not, and (3) between survivors and reference persons contributing to unique follow-up time.

We determined the validity of the registered mortality (GBA) and hospitalization (LMR) data by quantifying the agreements with observed deaths and hospitalizations for invasive cancer surgery in our available cohort data respectively. Although hospital admissions are not routinely recorded in our clinical database, there is data on surgical cancer treatment during the course of disease. We defined dates of invasive surgery (requiring hospitalization) for survivors who were surgically treated for primary cancer or recurrence between 1995 and 2005 according to our cohort data (=reference standard) and determined whether these hospitalizations were also identified through linkage with LMR at the same day and within 30 days of the date of surgery. Deaths were also recorded in the clinical database (=reference standard) and we determined if these deaths were registered in the same month in GBA (the day of the date of death was not available).

## Results

### Linkage of the cohort to GBA

**Fig 1:**
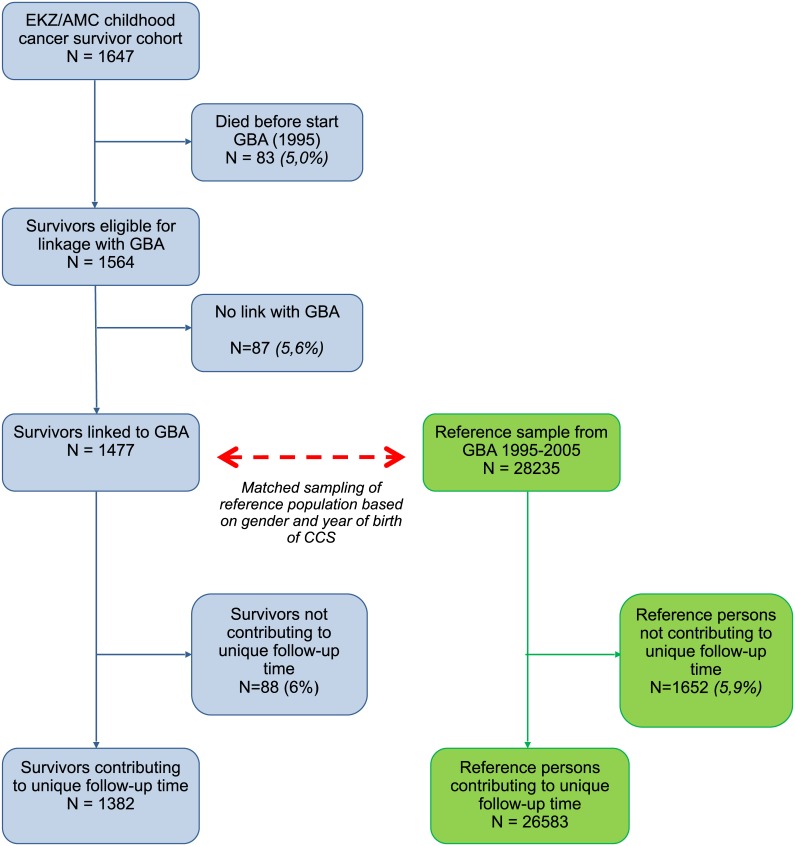
Flowchart of patients included in the EKZ/AMC cohort of childhood cancer survivors and sampled reference population from the GBA. Abbreviations: GBA: Dutch acronym for Municipal Personal Records Database; EKZ/AMC: Emma Children’s Hospital/Academic Medical Center

Fig 1 shows the flowchart of persons included in this study. Within the original cohort of 1647 survivors, 83 died before 1995 and were thus excluded from this study. The large majority (90%) of these 83 survivors had died in relation to a recurrence of their primary cancer.

Linkage step 1 of the study cohort to GBA was attempted for 1564 survivors. In 607 (39%) survivors the unique BSN was available and 604 of those (99.5%) were linked to GBA. The three other survivors with BSN were found to be valid, but not registered in GBA during the study period.

In the remaining 957 survivors, gender, date of birth and at least one postal code was available in 954 (99.7%). We had two reference dates with a postal code available in 659 (69%) and one postal code in 278 (18%) survivors. In 17 survivors the only postal code available was registered in our cohort before 1995 and we were thus not aware if this postal code would still be valid in 1995 or later. Of these 17 persons, 5 were retrieved within GBA.

Overall, 866 of 957 (90%) were linked based on date of birth, gender and postal code: 789 (82%) based on the postal code of the first reference date and 77 (8%) based on the second reference date available.

Distributions of characteristics in the original cohort, the eligible cohort (i.e. alive in 1995) and linked survivors are listed in Table 1. There were no differences in patient, cancer and treatment characteristics between these three groups.

**Table 1:** Characteristics of childhood cancer survivors, reference persons and subgroups based on the two-step medical record linkage process.

Childhood cancer survivors										Reference persons			
		Complete survivor (cohort n = 1647)		Survivors eligible for linkage (alive in 1995) (n = 1564)		Survivors linked to GBA (n = 1477)		Survivors contributing to unique follow-up time (n = 1382)		Reference sample from GBA (n = 28255)		Reference persons contributing to unique follow-up time (n = 26583)	
Clinical characteristic		n	%	n	%	n	%	N	%	n	%	n	%
Gender	Male	905	54.9	860	55.0	801	54.2	738	53.4	15298	54.1	14347	54.0
	Female	742	45.1	704	45.0	676	45.8	644	46.6	12957	45.9	12236	46.0
Year of birth	1954–1969	281	17.1	235	15.0	216	14.6	205	14.8	4268	15.1	4066	15.3
	1970–1985	937	56.9	900	57.5	847	57.3	819	59.3	15965	56.5	15462	58.2
	1986–1999	429	26.0	429	27.4	414	28.0	358	25.9	8002	28.3	7055	26.5
Type of inhabitant	Native inhabitant	na		na		1227	83.1	1148	83.1	20478	72.5	19461	73.2
	Non-native inhabitant	na		na		250	16.9	234	16.9	7757	27.5	7122	26.8
	First generation	na		na		47	3.2	44	2.3	4524	16.0	4137	15.6
	Second generation	na		na		203	13.7	190	13.7	3232	11.4	2984	11.2
Year of primary cancer diagnosis^a^	1966–1974	166	10.1	133	8.5	122	8.3	117	8.5	2411	8.5	2309	8.7
	1975–1984	550	33.4	508	32.5	479	32.4	464	33.6	9199	32.6	8932	33.6
	1985–1994	611	37.1	603	38.6	561	38.0	529	38.3	10563	37.4	10037	37.8
	1995–1999	320	19.4	320	20.5	315	21.3	272	19.7	6062	21.5	5305	20.0
Age at diagnosis^a^	Median (range)	5.9	0–17.8	6.8	0–17.8	5.8	0–17.8	6.1	0–17.8	5.9	0–18.4	6.0	0–18.4
	0–4 yr	723	43.9	692	44.2	653	44.2	607	43.9	12359	46.5	11518	43.3
	5–9 yr	445	27.0	416	26.6	395	26.7	364	26.3	7620	28.7	7197	27.1
	10–14 yr	372	22.6	351	22.4	334	22.6	318	23.0	6433	24.2	6118	23.0
	15–18 yr	107	6.5	105	6.7	95	6.4	93	6.7	1823	6.9	1750	6.6
Primary childhood cancer diagnosis	Leukemia/ lymphoma	740	44.9	697	44.6	671	45.4	624	45.2	na		na	
	CNS tumor	123	7.5	112	7.2	102	6.9	98	7.1	na		na	
	Sarcoma	310	18.8	292	18.7	280	19.0	269	19.5	na		na	
	Other solid tumors	370	22.5	361	23.1	388	26.3	356	25.8	na		na	
	Other and unspecified cancers	42	2.6	40	2.6	36	2.4	35	2.5	na		na	
Specific cancer treatments^b^	Anthracyclines	687	41.7	655	41.9	631	42.7	586	42.4	na		na	
	Alkylating agents	823	50.0	783	50.1	752	50.9	700	50.7	na		na	
	Other chemotherapy	440	26.7	417	26.7	390	26.4	364	26.3	na		na	
	Radiotherapy to head and/or neck region	454	27.6	404	25.8	392	26.5	374	27.1	na		na	
	Radiotherapy to thoracic and/or abdominal region	356	21.6	336	21.5	321	21.7	302	21.9	na		na	
	Radiotherapy to extremities	118	7.2	109	7.0	103	7.0	92	6.7	na		na	

Abbreviations: GBA: Dutch acronym for Municipal Personal Records Database; na: not applicable

^a^ For reference persons: date of cancer diagnosis and age of corresponding childhood cancer survivor.

^b^ All cancer treatment given before the date of five-year survival was included.

### Sampling of a reference population

Based on 1477 survivors, we sampled 28255 eligible reference persons from the general Dutch population, based on gender and year of birth (Fig 1). The distribution of non-native inhabitants (i.e. individual or one or both of the parents not born in the Netherlands) was lower in the survivor group than in the reference population (16.9% versus 27.5%, Table 1). This difference was primarily based on a difference between first-generation non-native inhabitants (19% of non-native survivors versus 58% of non-native reference persons).

### Linkage of the cohort and reference persons between GBA and LMR

The starting point in the second linkage step were 1382 (94%) out of 1477 survivors identified within GBA. Based on date of birth, gender and four number postal code, these 1382 survivors contributed to unique follow-up time in our study and thus had potential hospitalizations available from LMR. Of 28255 reference persons from GBA, 26583 (94%) contributed to unique follow-up time based on date of birth, gender and postal code.

The 94% survivors and 94% reference persons contributing to unique follow-up had similar distributions of year of birth, gender and calendar period (Table 1). There was no difference in patterns of loss due to non-uniqueness between survivors and reference persons. Specifically, loss of individuals due to non-uniqueness did not seem to be related to being a non-native inhabitant or to year of birth in either group. Table 2 shows the total potential and total unique follow-up time of survivors and reference persons. Median proportions of total unique follow-up time out of total potential follow-up time were 87% in survivors and 83% in reference persons identified in GBA (Table 2).

**Table 2:** Potential follow-up time and unique follow-up time of childhood cancer survivors and reference persons.

	Childhood cancer survivors			Reference persons		
	Sum	Median	Range	Sum	Median	Range
Potential follow-up time (years)	14983.9	11.0	0.1–11.0	292234.6	11.0	0.0–11.0
Unique follow-up time (years)	10645.6	8.8	0.1–11.0	194208.7	8.1	0.0–11.0
Unique follow-up time/potential follow-up time (%)		87	1–100		83	0.1–100

### Validity of mortality and hospitalization data

Between 1995 and 2005, 61 survivors died according to our cohort database. Of these 61 survivors, 55 (90%) were uniquely identified in GBA. Only one of 55 deaths was not registered in GBA. All registered deaths in GBA were in the same calendar month as the date of death registered in our cohort study. Thus, agreement of death registration within survivors registered to GBA was 98% (54 out of 55).

We selected 195 surgical hospitalizations in 156 survivors from our cohort database between 1995 and 2005 based on type of surgery (for a complete list of types of surgery, please contact the authors), of which 155 (81%) hospitalizations in 126 (82%) survivors occurred during unique follow-up time based on date of birth, gender and postal code. Of these 155 hospitalizations during unique follow-up time, 145 (94%) were registered in LMR at the exact date and 153 (99%) within 30 days of the date we registered the hospitalization in our study (Table 3).

**Table 3:** Agreement between the cohort register and LMR of 195 selected surgical hospitalizations within 156 childhood cancer survivors between 1995 and 2005.

Subgroup		Hospitalizations	%	Hospitalized persons	%
Eligible survivors		195		156	
Survivors linked to GBA		192	98%	153	98%
Survivors contributing to unique follow-up time		155	81%	126	82%
	Retrieved from LMR at same day	145	94%	121	96%
	Retrieved from LMR<30 days	153	99%	125	99%

Abbreviations: GBA: Dutch acronym for Municipal Personal Records Database; LMR: Dutch acronym for Hospital Discharge Register

## Discussion

This study shows that it is feasible and valid to study hospitalizations and mortality over time in a clinical cohort using a two-step medical record linkage approach, linking a clinical cohort of survivors of childhood cancer and two Dutch administrative registers. Only 1–2% of information about deaths and hospitalizations in traced survivors of childhood cancer was not retrieved using this approach. We described the key technical steps of the linkage procedure and highlighted specific pitfalls in these types of studies. In this section we will further elaborate on these pitfalls and give recommendations for future cohort studies based on record linkage.

### Left-truncated data

Many record linkage studies face the issue that the registers containing longitudinal outcome data start at a specific point in calendar time. In our situation, linkage was only possible from 1995 onwards. The outcome of interest could have occurred before 1995 in part of the cohort, during which linkage was not possible. Our outcome data are therefore left-truncated, i.e. we do not know if the event occurred before the start of the study. For longitudinal assessment of hospitalizations, estimates will still be valid if individuals are only included during the period in which outcomes could be assessed and the entry-time is taken into account [16].

### Initial loss of persons through linkage with national population register

There are several reasons why patients from a study cohort have to be excluded from the linkage study in the initial linkage step, including missing values, data entry error on linking variables or multiple persons having the same values on all linking variables (administrative twins). In our study, reasons for non-linkage could be missing postal codes (n = 3), most likely because individuals were living abroad, or postal codes only available before 1995 (n = 17). It is also possible that a different address was registered in our cohort study compared to the official address registered in GBA. This occurs more frequently in young adults [17]. Such an initial loss of patients reduces the power of a study and can pose a threat to the generalizability of linkage results.

A very high proportion of our cohort could be linked to GBA. Especially linkage based on the unique BSN yielded a 99.5% linkage. Linkage based on gender, date of birth and postal code at a reference date (82%) was good, and adding a postal code at an extra reference date increased the linkage to 90% based on these three identifiers. We used a postal code with four numbers and two letters but previous research shows that using four numbers of the postal code would not largely affect initial linkage success [6]. If there is informative non-linkage in a study, i.e. if linked persons have a different prognosis than persons not linked, the generalizability decreases and there is a risk of selection bias. However, if this difference can be explained by characteristics available in the cohort (e.g. age, gender, cancer treatment, calendar year of treatment), analyses conditional on a specific characteristic will still be valid. In addition, within this study we showed that patient characteristics of the eligible cohort and the linked cohort were comparable. An important strength of the Dutch situation is that through linkage with GBA there is up-to-date, high quality recording of all deaths and thus low risk for on-going contribution to person-time after a death that was not registered.

### Loss of persons and unique follow-up time through linkage between administrative registers in the absence of a unique person identifier

In studies with linkage between administrative registers not based on a unique person identifier, there will be loss of persons who were never unique during the study period based on the available linkage parameters. In addition, there will be loss of unique follow-up time and loss of information about the outcome of interest when persons are temporarily non-unique. In our study, reasons for (temporary) non-uniqueness due to administrative twins could be the play of chance, a true twin [12] or a large number of people living in an area with the same four number postal code. Although not found in this study, this last reason is more common among students, elderly and disabled. Also, immigrants more often have the same date of birth registered than expected, mainly because they are more often registered as born on the first of January or July [6]. Linkage between the two databases in our study was based on four number postal code. Using a full postal code would slightly increase linkage successes and thus unique periods [14]. However, full postal code is not always available in LMR records and therefore precludes longitudinal studies on hospitalization.

When loss of persons and/or unique follow-up time is high, this could potentially lead to low generalizability, power issues and several types of bias.

In our study a similar and high proportion (94%) of survivors and reference persons contributed to unique follow-up time based on gender, date of birth and postal code. The median percentages of unique follow-up time in relation to potential maximum follow-up time was high and in line what could be expected based on the percentage of persons in the Netherlands with a unique combination of gender, date of birth and postal code (84%) [10,16]. Furthermore, the percentage unique follow-up time was comparable between the cohort of survivors and members of the reference population.

In addition, matching characteristics of survivors and reference persons contributing to unique follow-up time were comparable. The loss of survivors due to non-uniqueness therefore seemed unrelated to being a survivor, thereby reducing the risk of differential misclassification (i.e. systematic differences in outcome between study cohort and reference persons).

Except for the proportion of non-native inhabitants, survivors and reference persons had comparable distributions of characteristics after excluding non-unique persons and non-unique follow-up time. As first generation non-native inhabitants were defined as individuals born in another country who subsequently immigrated to the Netherlands, it is explainable that in a population of childhood cancer survivors (diagnosed with cancer and thus selected for our cohort at age 6 on average) the proportion of first generation non-native inhabitants is lower than in a reference population within this study (sampled from the general Dutch population at age 25 on average, with 19 years extra to immigrate). We recommend future studies to consider immigrant status in the sampling of reference persons. Alternatively, studies should assess the effect of a lower proportion of non-native inhabitants in a multivariate model. The consequence of non-unique follow-up time is that hospitalizations will be missed. This is not an issue, as long as the hospitalizations are presented in rates per unique follow-up time and if the risk of hospitalization is similar during unique and non-unique periods. The yield of hospitalized survivors of our cohort in LMR was 82% and thus comparable to the expected yield for the cohort’s age range (83–85%) [17].

Main reasons for absence of hospitalizations in LMR could be a hospitalization outside the Netherlands (this was not taken into account in the selection of surgical hospitalizations according to our cohort register) or in one of the few hospitals that did not participate in the LMR between 1995 and 2005. Due to hospitalizations abroad (of Dutch residents), there will be a slight underestimation of hospitalization rates. It is not unlikely that the hospitalization rates in survivors will be more affected by this loss than reference persons. Survivors are at risk for health problems in general, and second cancers specifically, and it is not uncommon that for complicated (cancer) treatment or treatment not (readily) available in the Netherlands, patients are hospitalized outside the country. It is also more likely that compared to the reference population, survivors have been hospitalized more frequently in the single cancer hospital within the Amsterdam area that does not register in LMR. Other reasons for missing hospitalizations in LMR could be administrative errors and other administrative reasons; persons who used another postal code for administrative reasons during the hospitalization or persons who did not register a new address to GBA yet. Future studies should be aware of such (small) effects on hospitalization rate.

A more serious problem in record linkage is when hospitalizations or death are missed when a person is unique. In our study, we only encountered the absence of one death in GBA. This could be due to emigration or because a person changed address but did not register the new address. Such record linkage errors directly lead to an underestimation of hospitalization risks due to immortal follow-up time after the death if it remains unnoticed. However, we showed high sensitivity of death and hospitalization registration. The risk of such type of information bias was thus low.

A final problem in record linkage studies could be when a hospitalization is linked to a wrong individual. Although we were not able to study this potential threat, another study has shown that this risk is very low in the Dutch situation [14].

### Recommendations for future studies

Our study demonstrated that Dutch cohort studies can obtain valid hospitalization and mortality data over time from two nationwide administrative registers in absence of one unique identifier.

A prerequisite for such studies using a two-step medical record linkage approach is a high linkage proportion of cohort members to GBA. It is therefore recommended to retrieve the BSN for cohort patients, taking into account the ethical and legal aspects in each specific situation. Alternatively, cohort studies should collect complete information about date of birth, gender, postal code and date of death if applicable. A postal code at a second reference date increases linkage success.

There are excellent opportunities to sample an appropriate reference population from GBA with which to compare hospitalization rates. In addition, hospitalization registration in LMR is of good quality. Some events are lost due to non-uniqueness of persons, but estimation of admission density is valid when also the non-unique follow-up time is excluded. Another important methodological issue to take into account for the Dutch situation is left-truncation, as there is only hospitalization data electronically available from 1995 onwards. Finally, we recommend assessing characteristics of the initial cohort and the linked cohort, in order to define the generalizability of the studied cohort and characteristics that need to be adjusted for in multivariate analyses.

With the increasing possibilities of record linkage to administrative data, it is essential that future studies using medical record linkage protect the disclosure of individual data and take into account the legislation to protect individual privacy. This paper provides information on this, but the matter is complex and changes over time [18]. We therefore additionally recommend to consult a local medical ethics expert to assess legal and ethical matters per study [15].

## Conclusion

In summary, there is great potential for cohort studies to study hospitalizations and mortality over long periods of time using a two-step medical record linkage approach with nationwide registers. Cohort studies in the Netherlands should take our recommendations of a two-step medical record linkage approach into account. Future studies will be able to examine hospitalization rates or other clinical events longitudinally (e.g. to study absolute incidence rates or prognostic factors) and can thereby contribute to broad research possibilities with insights into the burden of clinical events and healthcare use allowing improvement in future patient care.

## Supporting Information

S1 Fig Graphical representation of the two-step medical record linkage process, linking a study cohort with (1) GBA and (2) LMR in order to retrieve hospitalization data from LMR. Abbreviations: GBA: Dutch acronym for Municipal Personal Records Database; LMR: Dutch acronym for Hospital Discharge Register.
(EPS)

S1 Methods Legal and logistics aspects of medical record linkage with Statistics Netherlands (DOCX)
