# Supplementary material for: Studying Hospitalizations and Mortality in the Netherlands: Feasible and Valid Using Two-Step Medical Record Linkage with Nationwide Registers
Source: PLoS One. 2015 Jul 6;10(7):e0132444. doi: 10.1371/journal.pone.0132444 (PMC4493069; doi:10.1371/journal.pone.0132444)
Supplement: S1 Methods — (DOCX) [file pone.0132444.s002.docx]

**Supporting information**

**S1 Methods**. **Legal and logistics aspects of medical record linkage with Statistics Netherlands**

Statistics Netherlands endorses the Code of Practice for European Statistics with the Statistics Netherlands Act that covers, amongst others, statistical confidentiality (1, 2). They provide a secure environment with a remote access facility that facilitates physical and legal protection of data (3, 4). Statistical confidentiality is maintained by ensuring that patient individual data are not revealed. All researchers involved in the current project were approved by Statistics Netherlands and they signed a confidentiality agreement. Statistics Netherlands data is not freely accessible to the public and costs are involved for setting up a research project and we had to cover the costs of record linkage. Costs vary depending on the number of researchers and dataset topics, starting at 4000 euros for medium sized projects of 3 to 4 researchers.

References

1. Quality declaration of Statistics Netherlands. The Hague/Heerlen: Statistics Netherlands; 2014.

2. Statistics Netherlands (CBS). Act of 20 November 2003 (Statistics Netherlands Act) 2003 [9 April 2015]. Available from: <http://www.cbs.nl/NR/rdonlyres/BBD8113D-7EE5-4BE4-8879-685253B31882/0/statisticsnetherlandsactjune2013.pdf>.

3. Microdata services: conduct your own research using data from Statistics Netherlands: Statistics Netherlands; [9 April 2015]. Available from: <http://www.cbs.nl/en-GB/menu/informatie/beleid/zelf-onderzoeken/default.htm?Languageswitch=on>.

4. Requirements for Remote Access PC: Statistics Netherlands; [9 April 2015]. Available from: <http://www.cbs.nl/NR/rdonlyres/79C1D4A9-07B9-4785-9D16-6198653541CE/0/130625requirementsrapc.pdf>.
